# A case of cardiac sarcoidosis with a ventricular aneurysm visualized by four-dimensional left ventricular imaging using TrueVue glass

**DOI:** 10.1093/ehjimp/qyag011

**Published:** 2026-02-17

**Authors:** Yusuke Nakashima, Michio Yamada, Ayumi Omuro, Shinichi Okuda, Motoaki Sano

**Affiliations:** Department of Cardiology, Hagi Civil Hospital, 3460-3 Tsubaki, Hagi, Yamaguchi 758-0061 Hagi, Japan; Division of Cardiology, Department of Medicine and Clinical Science, Yamaguchi University Graduate School of Medicine, 1-1-1 Minami-Kogushi, Ube, Yamaguchi 755-8505, Japan; Department of Cardiology, Hagi Civil Hospital, 3460-3 Tsubaki, Hagi, Yamaguchi 758-0061 Hagi, Japan; Division of Cardiology, Department of Medicine and Clinical Science, Yamaguchi University Graduate School of Medicine, 1-1-1 Minami-Kogushi, Ube, Yamaguchi 755-8505, Japan; Department of Cardiology, Hagi Civil Hospital, 3460-3 Tsubaki, Hagi, Yamaguchi 758-0061 Hagi, Japan; Division of Cardiology, Department of Medicine and Clinical Science, Yamaguchi University Graduate School of Medicine, 1-1-1 Minami-Kogushi, Ube, Yamaguchi 755-8505, Japan; Department of Clinical Laboratory Science, Faculty of Health Sciences, Yamaguchi University Graduate School of Medicine, 1-1-1 Minami-Kogushi, Ube, Yamaguchi 755-8505, Japan; Division of Cardiology, Department of Medicine and Clinical Science, Yamaguchi University Graduate School of Medicine, 1-1-1 Minami-Kogushi, Ube, Yamaguchi 755-8505, Japan

A 65-year-old woman underwent percutaneous coronary intervention for a mid-left anterior descending artery lesion. Although no ventricular aneurysm (VA) was observed during the acute phase, follow-up echocardiography unexpectedly revealed a lateral wall VA. This location did not correspond to the expected ischaemic territory on CT (*Panel A*, top), indicating a diagnostic mismatch and prompting multimodality imaging. TrueVue Glass (Philips CVx system) was used to characterize this atypically-located VA. Compared with conventional 2D echocardiography, TrueVue Glass provided improved endocardial border delineation and enhanced spatial appreciation of aneurysmal morphology without contrast. Although not inherently diagnostic, it enabled flexible four-dimensional (4D) visualization resembling left ventricular angiography, allowing comprehensive structural assessment (*Panel B*, Supplementary data online, *Video S1*). FDG-PET/CT demonstrated focal left ventricular uptake (*Panel A*, middle), and cardiac MRI confirmed late gadolinium enhancement (LGE) involving the aneurysmal region (*Panel A*, bottom). Follow-up coronary angiography showed no restenosis or new lesions, and left ventriculography identified a true VA in LV segment 7 according to the AHA 17-segment model (see Supplementary data online, *Video S2*), consistent with TrueVue findings (*Panels C and D*, Supplementary data online, *Video S3*).

Based on the 2016 Japanese Circulation Society criteria—including the presence of a VA, reduced LV systolic function, localized FDG uptake, and LGE on MRI—the patient was diagnosed with cardiac sarcoidosis. TrueVue Glass, originally developed for foetal imaging, has recently been adapted for cardiac structural assessment. In this case, its non-invasive, real-time 4D visualization provided superior spatial understanding of the VA compared with 2D echocardiography, supporting the diagnosis of cardiac sarcoidosis.

**Figure:**
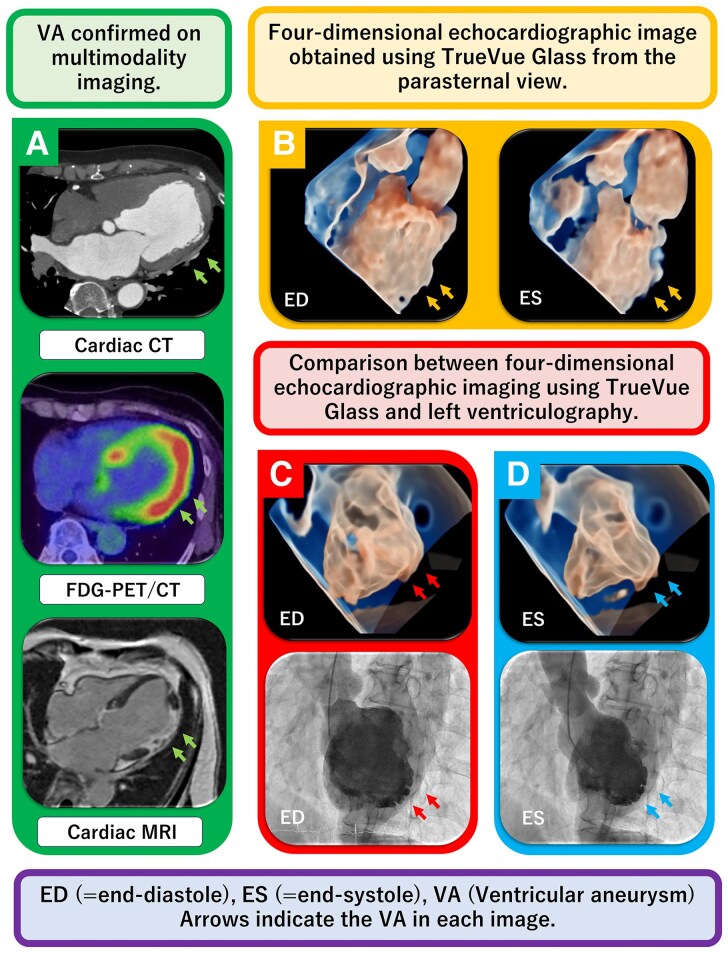


## Supplementary Material

qyag011_Supplementary_Data

## Data Availability

Data sharing is not applicable to this article as no datasets were generated or analysed during this study.

