# Supplementary figures and images for: A case of cardiac sarcoidosis with a ventricular aneurysm visualized by four-dimensional left ventricular imaging using TrueVue glass
Source: Eur Heart J Imaging Methods Pract. 2026 Feb 17;4(1):qyag011. doi: 10.1093/ehjimp/qyag011 (PMC12988775; doi:10.1093/ehjimp/qyag011)

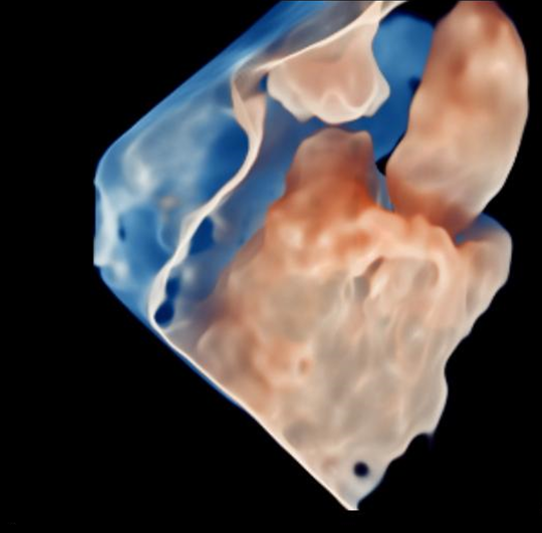

Supplement: qyag011_Supplementary_Data [file qyag011_supplementary_data.zip › Video1 still image.png]

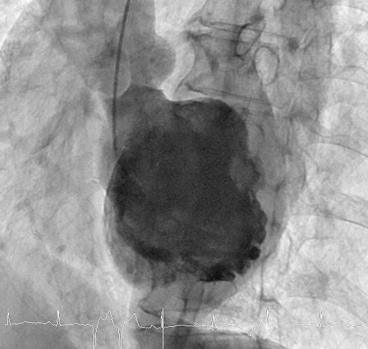

Supplement: qyag011_Supplementary_Data [file qyag011_supplementary_data.zip › Video2 still image.png]

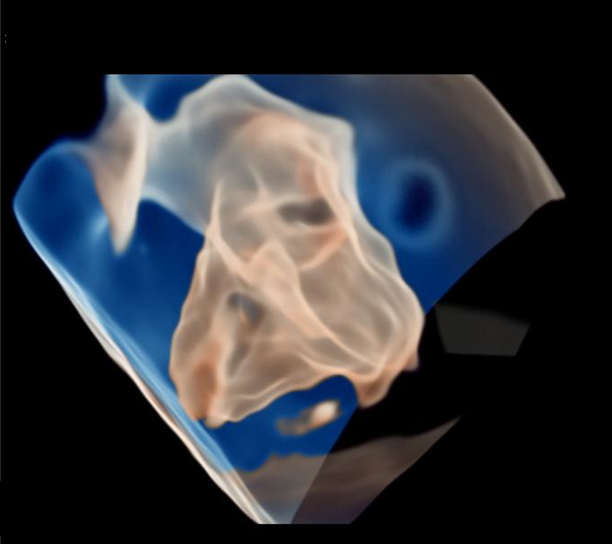

Supplement: qyag011_Supplementary_Data [file qyag011_supplementary_data.zip › Video3 still image.png]
